# Supplementary material for: Analysis of Transcriptional Changes in Different Brassica napus Synthetic Allopolyploids
Source: Genes (Basel). 2021 Jan 11;12(1):82. doi: 10.3390/genes12010082 (PMC7827416; doi:10.3390/genes12010082)
Supplement: Supplementary file 1 [file genes-12-00082-s001.zip › Additional file 4.docx]

Additional file 4: GO enrichment analysis of differential genes aligned with the AA\CC genome.

| /AA | Description | FDR |
| --- | --- | --- |
| AACC1 | metabolic process | 0.006029 |
|  | oxidoreductase activity | 0.006469 |
|  | carbohydrate metabolic process | 0.02029 |
| AACC2 | response to auxin | 0.002629 |
|  | response to chemical | 0.019114 |
|  | carbon-nitrogen lyase activity | 0.019114 |
|  | response to hormone | 0.019114 |
| AACC3 | carbohydrate metabolic process | 0.008613 |
| AACC4 | response to chemical | 0.000598 |
|  | response to hormone | 0.00516 |
|  | response to auxin | 0.00516 |
|  | response to organic substance | 0.00516 |
|  | response to endogenous stimulus | 0.00516 |
| AACC5 | response to auxin | 0.001926 |
|  | response to chemical | 0.001926 |
|  | response to hormone | 0.00229 |
|  | response to endogenous stimulus | 0.002518 |
|  | response to organic substance | 0.00457 |
| AACC6 | oxidoreductase activity | 0.000157 |
|  | metabolic process | 0.00315 |

| /CC | GO term | FDR |
| --- | --- | --- |
| AACC1 | ribosome | 4.38E-41 |
|  | structural constituent of ribosome | 4.38E-41 |
|  | ribonucleoprotein complex | 1.18E-35 |
| AACC2 | single-organism biosynthetic process | 1.43E-06 |
|  | organonitrogen compound metabolic process | 5.01E-06 |
|  | organonitrogen compound biosynthetic process | 1.03E-05 |
| AACC3 | biological_process | 8.40E-05 |
|  | single-organism biosynthetic process | 8.40E-05 |
|  | organonitrogen compound metabolic process | 8.40E-05 |
| AACC4 | organonitrogen compound metabolic process | 6.84E-07 |
|  | single-organism biosynthetic process | 4.28E-06 |
|  | organonitrogen compound biosynthetic process | 4.28E-06 |
|  | single-organism metabolic process | 7.45E-06 |
| AACC5 | single-organism biosynthetic process | 1.19E-05 |
|  | single-organism metabolic process | 1.19E-05 |
| AACC6 | single-organism biosynthetic process | 6.87E-05 |
|  | ribonucleoprotein complex | 0.001324 |
| AACC7 | ribosome | 1.14E-31 |
|  | structural constituent of ribosome | 1.14E-31 |
|  | ribonucleoprotein complex | 7.25E-28 |
|  | organonitrogen compound metabolic process | 6.10E-23 |
|  | organonitrogen compound biosynthetic process | 4.27E-22 |
| AACC8 | organonitrogen compound biosynthetic process | 2.78E-07 |
|  | organonitrogen compound metabolic process | 2.78E-07 |
|  | ribosome | 2.78E-07 |
|  | ribonucleoprotein complex | 2.78E-07 |
|  | structural constituent of ribosome | 2.78E-07 |
